# Does corporate social responsibility impact equity risk? International evidence

**DOI:** 10.1007/s11156-022-01059-7

**Published:** 2022-04-06

**Authors:** Alice Monti, Pierpaolo Pattitoni, Barbara Petracci, Otto Randl

**Affiliations:** 1 Risk Management Unit, UnipolSai Assicurazioni S.p.A., Bologna, Italy; 2 Department of Statistical Sciences “Paolo Fortunati”, University of Bologna, Bologna, Italy; 3 Department of Management, University of Bologna, Bologna, Italy; 4 Department of Finance, Accounting and Statistics, WU Vienna University of Economics and Business, Vienna, Austria

**Keywords:** Corporate social responsibility, Equity risk, International evidence, G32, M14, O57

## Abstract

Based on a large panel of listed firms from 52 countries in the period 2002–2020, we investigate the relationship between corporate social responsibility (CSR) and equity risk. We confirm previous evidence that higher CSR scores are related to lower risk measures, considering all types of risks: total, systematic, and idiosyncratic. Analyzing a large international sample allows us to investigate the role of country and company characteristics in the relationship between CSR scores and risk measures. The risk-reducing effect is more pronounced in weaker institutional environments. It is stronger in civil-law countries, in countries with low security regulation or disclosure requirement levels and where financial information is less widespread. Firms in high impact or high profile industries benefit more from CSR than firms in other industries as do firms that are not cross-listed. The financial crisis has increased the risk-reducing effect of CSR. The main results are confirmed in the COVID-19 period.

## Introduction

There is a growing interest among investors, corporate managers, and academics if and how environmental, social, and governance (ESG) criteria should be incorporated into portfolio construction. The 2018 Global Sustainable Investment Review (GSIA 2018) reports that assets managed under socially responsible investment (SRI) strategies amounted to $31 trillion in 2018, out of which $19.5 trillion made use of the integration of ESG factors. While the remarkable ESG focus in mutual fund management is a rather recent phenomenon, sophisticated investors such as sovereign wealth funds and university endowments have a long tradition in SRI strategies. A prominent example is the Norwegian Government Pension Fund Global with its clear and transparent SRI focus. In their report on the fund’s responsible investment strategy, Dimson et al. (2013) state as one of the principal motivating factors for CSR the fact that sustainability can enhance performance as it allows to avoid risks.

There are several channels through which CSR activities can potentially mitigate equity risk. First, CSR can lead to a reduction of operational risks related to litigation, regulatory intervention, and reputation (see, for example, Hong et al. 2019). Similarly, high-CSR firms are associated with higher employee job satisfaction (Edmans 2012), which likely lowers the risk of losing key personnel, thus increasing stability. While intuitively a reduction of operational risk should primarily be seen in measures for both idiosyncratic and total risk, for some drivers of operational risks an effect on systematic risk measures has also been discussed. Further, due to the nature of ESG-related risks that tend to be rare, large and non-diversifiable, incorporating ESG criteria into the investment process can protect investors from downside risk (Jagannathan et al. 2018). Second, the financial channel stemming from investor preferences is a potentially large driver of risk-reduction stemming from CSR. The classic reference here is Heinkel et al. (2001), who show that beyond any operational channel, with clearly pronounced investor preferences, clean firms will face lower cost of capital. This corresponds directly to a lower measure of systematic risk, beta.

**Fig. 1:**
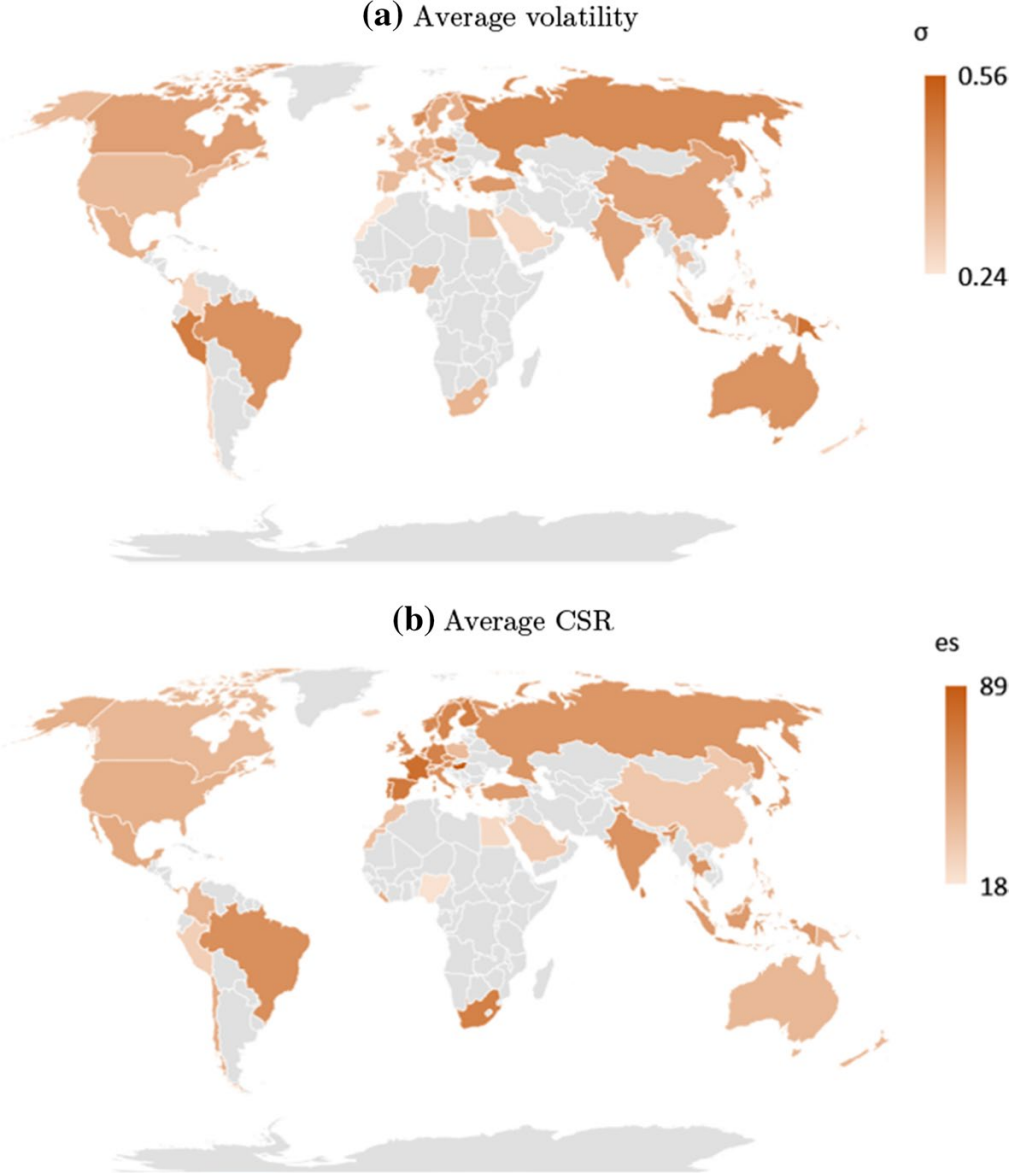
Volatility and CSR by country. Volatitility is the average standard deviation of stock returns by country. CSR is measured as the average of the environmental and social score from ASSET4 by country

The various channels for the negative CSR-risk relationship further suggest cross-sectional heterogeneity. CSR activities are likely to have stronger effects when environmental and social aspects matter more to investors, and if firms operate in a difficult institutional environment. As a motivating illustration, Fig. 1 shows the variation across countries both in terms of CSR and equity risk. The plots suggest that countries with high levels of CSR tend to have low stock market volatility. In our paper, we aim at understanding if such patterns in the distribution of CSR measures and risk measures can be understood from cross-country institutional differences, such as investor protection, disclosure requirements, the level of earnings management, and the frequency of interim reporting. Similarly, we investigate company characteristics and the time-dimension. Investors will pay more attention to CSR activities of the firms that belong to industries with a high impact on stakeholders. Further cross-sectional differences can be expected from a company access to international markets. As broadening the shareholder base is one explanation for the CSR-risk link, firms with already good access to foreign markets through equity cross-listings can be expected to benefit less from CSR in terms of lower risk measures. Some of the stated motives for CSR are insurance-like, in that they should protect against risk of rare but large events. Such insurance should be most valuable in turbulent times. We thus investigate if the magnitude of the negative CSR-risk relationship is more pronounced during and after the financial crisis than before.

In our empirical analysis, we go beyond beta and use risk measures that also capture total, idiosyncratic and downside risk. This approach is consistent with the view that there are systematic and unsystematic components of equity risk that can be affected in different ways by CSR activities. We use a large panel of listed firms from 52 countries for the periods 2002–2015, using Thomson Reuters ASSET4 (ASSET4) and 2016–2020 (using Refinitiv). While divergence of ESG scores across data providers has been documented by Berg et al. (2020), we believe that ASSET4 offers several advantages for our study compared to other databases. First, it has worldwide coverage and uses selection rules that are designed to mitigate a potential sample selection bias. Second, Berg et al. (2020) show that ASSET4 is among the major data providers with scores highly correlated in the order of magnitude of 0.6–0.8 with the scores of the other data providers (RobecoSAM, Sustainalytics, Vigeo-Eiris), while the scores of KLD exhibit the lowest correlations with all other raters. Using our large, international cross-section of firms, we confirm previous studies that find a negative CSR-risk relationship mostly on smaller sets of firms and risk indicators (e.g., Oikonomou et al. 2012; Cai et al. 2016; Albuquerque et al. 2019; Hoepner et al. 2021). The above mentioned studies, with the exception of Hoepner et al. (2021), focus only on US firms (as they use KLD as ESG data provider); while Hoepner et al. (2021) focus only on 573 worldwide firms (using a proprietary database).

Our main contribution to the literature is twofold. First, we provide strong evidence that institutional factors matter for the strength of the negative link between CSR and risk. In a nutshell, the weaker the institutions, the more pronounced the negative relationship. So far, the literature has provided little evidence on the role of the institutional framework on the CSR-risk relationship. Closest to our analysis in this respect are the findings by Dhaliwal et al. (2014) that the relationship between CSR disclosure and the cost of equity is stronger in countries that are more stakeholder oriented and exhibit higher levels of financial opaqueness, and Benlemlih and Girerd-Potin (2017) who find that CSR affects risk only in more stakeholder-oriented (thus less shareholder-oriented) countries. In our paper, we provide evidence that the effect is stronger in countries with less stringent security regulation, low levels of disclosure requirements, and where financial information is less widespread. With respect to the legal origin, the relationship between CSR and risk is stronger in civil-law countries than in the more investor-friendly common law countries. Second, we provide strong evidence that firm characteristics matter for the strength of the CSR-risk link. Previous work has analyzed the relationship between company characteristics and the level and disclosure of CSR.^1^ We add to the understanding of the importance of these characteristics for CSR by providing evidence on the CSR-risk link. Firms in industries with a high impact on stakeholders or a high profile – i.e., controversial industries – benefit more from CSR than firms in other industries. Similarly, the risk-reducing effect of CSR is higher for firms with no or a low number of international cross-listings. These firms are not yet subject to the more stringent monitoring of another stock exchange, so ESG scores matter more. We observe a clear time pattern: CSR activities have developed into more effective instruments to reduce equity risk since the financial crisis. Finally, we provide evidence that our main results carry over to the COVID-19 period.

Our findings are important for corporate managers, investors, and policymakers. First, our results suggest that CSR activities should become a core element of a firm business rather than stand-alone programs, as they seem to be able to reduce risk. Second, our results clearly show that the effectiveness of CSR in reducing risk is specific to the institutional setting. The benefits of CSR activities are more pronounced for companies based in weak institutional environments and active in controversial industries. For such firms, investors should, therefore, particularly pay attention to ESG scores. Since institutions are mostly controlled by policymakers, the latter may adopt policies to encourage socially responsible actions and benefit from positive environmental and social externalities.

The remainder of the paper is structured as follows. Section 2 discusses the literature and develops the hypotheses. Section 3 describes our data sources, variables, and the empirical methods, while Sect. 4 discusses our results. Section 5 concludes.

## Why does CSR impact equity risk?

The most direct channel from CSR activities to stock price fluctuations is the effect on the riskiness of a company’s operations. According to Jagannathan et al. (2018), the risk of adverse affects from sudden changes in regulation and consumer behavior can be mitigated by the implementation of CSR activities. Socially irresponsible firms often face higher litigation, lawsuit, regulatory intervention, and reputational risks. Hong and Kacperczyk (2009) provide evidence that litigation risk is an important driver of the higher risk premia of the so-called sin stocks—companies involved in producing alcohol, tobacco, and gaming. More recently, Hong et al. (2019) find that CSR activities mitigate legal risks, as firms perceived as socially responsible face lower fines for violations of the Foreign Corrupt Practices Act. CSR can also reduce the riskiness of firms’ cash flows by serving as a product differentiation strategy. Albuquerque et al. (2019) find high-CSR firms to have lower systematic risk, in particular those firms with a low price elasticity of demand. In the context of employees’ job satisfaction, Edmans (2012) finds a positive impact from CSR on stock returns. While he states that risk reduction is a potential advantage of CSR and controls for firm risk in his regressions, his analysis does not formally test for an effect from CSR on risk. Most types of operational risk that are likely mitigated through CSR activities show up as idiosyncratic risk. With increased consumer attention towards environmental and social criteria, improved resilience on product markets might translate into lower exposure to some systematic risk.

Investors and their preferences are the second channel through which CSR potentially impacts equity risk, as engagement in CSR activities broadens a firm’s shareholder base. Heinkel et al. (2001) have first analyzed how investors can induce management to turn a company green, even if this is costly from an operational viewpoint. In their model, investors can buy shares from two types of firms: clean and polluting. When a sufficient number of investors shuns polluting firms, limited risk-sharing leads to a low (high) cost of capital for clean (polluting) firms. Hence, operational benefits are not needed to turn CSR into a value-maximizing strategy for some firms. In the context of our study, the reduction in the cost of capital will translate into a lower measure of systematic risk, beta. Riedl and Smeets (2017) provide empirical evidence that investors in socially responsible mutual funds willingly accept to earn lower returns as they put their social preferences first in the investment decisions. Lower exposure to systematic risk driven by investor preferences might alternatively be interpreted as mispricing. Fama and French (2007) discuss how misinformed investors or investors with specific tastes can distort market prices. Empirically, Galema et al. (2008) find that SRI results in lower book-to-market ratios. Not only CSR attracts socially responsible investors, but these investors might provide a more stable shareholder base. Bollen (2007) and Renneboog et al. (2011) find ethical money to be less sensitive to past negative returns than conventional fund flows. Potentially, this might be associated with lower stock price volatility. Recently, Pastor et al. (2020) build on Heinkel et al. (2001) to establish a distinct ESG risk factor. In their model, the market betas of green stocks depend on the correlation between a macro factor and the ESG factor. Oikonomou et al. (2012) argue that if CSR has insurance-like effects and returns are asymmetrically distributed, CSR strategies should have a stronger effect on downside and extreme risk measures than standard ones. Empirically, Hoepner et al. (2021) provide evidence that CSR shareholder engagement reduces downside and extreme risks, measured using lower partial moments of the return distribution and Value-at-Risk. Ilhan et al. (2020) provide similar evidence for firms with high carbon emissions. The above discussion suggests a negative relationship between CSR and risk. Furthermore, it points towards the importance of considering different measures for risk, such as total risk, systematic and idiosyncratic risk, and downside risk.^2^

While we proceed in our empirical analysis to confirm the negative CSR-risk relationship, we expect cross-sectional heterogeneity. CSR activities are likely to have stronger effects the more environmental and social aspects matter, and if firms operate in a difficult institutional environment. Our hypotheses, therefore, focus on the contingent aspects of the CSR-risk relationship. Country, industry, and firm characteristics as well as special periods like the recent financial crisis and the COVID-19 period are likely to influence the relationship between CSR and equity risk.

La Porta et al. (1998) have kick-started a vast amount of contributions that help understand how the legal environment and shareholder protection shape corporate finance decisions and capital markets. A beneficial effect of CSR on risk-reduction can be expected to be more pronounced in weak institutional environments, where the legal framework does not sufficiently protect investors, such that CSR will enhance corporate standards. We expect this to be the case for civil-law countries, that are identified by La Porta et al. (1998) as less shareholder-friendly than common-law countries. The effect should be more pronounced for idiosyncratic risk measures (which are likely driven by operational risk drivers) than for systematic risk (that will partly mirror investor preferences). Measurement through the legal origin alone might be crude, as the legal environment has many facets. We expect stronger effects from CSR on risk in countries with low levels of security regulation, weak disclosure requirements, high levels of earnings management and low interim reporting frequency. Some evidence from the existing literature indicates that the legal and institutional framework matters for CSR. Edmans et al. (2014) show that employee satisfaction depends on the legal environment and the institutional context. They find superior long-run returns, current valuation ratios, future profitability, and earnings surprises in countries with flexible labor markets (e.g., the US), but not ones with rigid labor markets where legislation already provides higher minimum standards (e.g., Germany). Jiraporn et al. (2014) illustrate the importance of local information for the link between CSR and credit ratings. Finally, Mitchell et al. (1997) show that the negative relationship between CSR disclosure and the cost of equity capital is stronger in stakeholder-oriented countries. These considerations lead us to the formulation of our first hypothesis as:

1. *The negative relationship between CSR and equity risk is more pronounced in civil law countries, countries with low standards of security regulation, low disclosure requirements, high levels of earnings management and a low frequency of interim reporting.*

If investors with CSR preferences care about the positive externalities from the implementation of CSR activities, they will look beyond country characteristics. A natural starting point is the industry classification. Cai et al. (2016) provide evidence for US firms on a negative CSR-risk relationship for manufacturing firms, while even a positive relationship for the service sector. More generally, we expect that risk mitigation will be strong for industries where the impact from CSR is high. Thus, we categorize firms according to the magnitude of their impact on stakeholders. Industries with a high impact on stakeholders are often referred to as controversial sectors, such as Basic Materials, Oil and Gas, and Utilities (Jackson and Apostolakou 2010). Similarly we group firms into high profile industries as defined by Roberts (1992) on the basis of high consumer visibility, a high level of political risk, and concentrated and intense competition. For many firms, CSR will not be the only way to broaden their shareholder base and seek risk-reduction. For example, a firm’s management can pursue a cross-listing on a foreign stock exchange. This gives companies easier access to capital, tends to increase the turnover of their stocks, and has positive spill-over effects on their product markets. On the other hand, a cross-listing necessitates compliance with the foreign exchange’s regulations (Pagano et al. 2002; Halling et al. 2008). While Boubakri et al. (2016) find that cross-listed firms on average engage in a higher level of CSR activities, they have not analyzed the effect of cross-listings on the CSR-risk relationship. We expect the gain in risk-reduction to be smaller for cross-listed firms. The reason is that both the cross-listing decision and CSR activities as individual measures are likely to increase the shareholder base. For an already large shareholder base, a further increase will have a smaller incremental effect. A similar argument can be made for the risk-reducing effect of accepting higher standards. In total, we have three company-specific indicators: the impact of industries, the profile of industries, and the existence of a cross-listing. For all three measures, the potential differences stem from both operational risks and investors’ preferences. Therefore, we expect systematic and idiosyncratic risk components to be similarly affected. We thus formulate:

H2: *The relationship between CSR and equity risk is contingent on firm characteristics. The negative relationship is more pronounced for firms from industries with a high impact on stakeholders and a high profile. It is less pronounced for firms which are cross-listed. *

Finally, insurance-like motives for CSR might be especially important in times of economic and financial crisis. Lins et al. (2017) emphasize that social capital helps build stakeholder trust and pays off more during low-trust periods. The authors find empirical evidence that firms with high levels of CSR recorded stock market outperformance during the financial crisis. In other words, their results suggest that investment in firm-specific social capital provides insurance in times of confidence crisis. Hence, we formulate our last hypothesis:

H3: *The relationship between CSR and equity risk is contingent on the financial-crisis phase. It will be more pronounced during and after the financial crisis than before.*

## Data and empirical methods

### Data

We combine and match data from several sources. We obtain firm-specific financial variables from Datastream. This includes daily stock returns that we use to calculate risk measures and data to construct the control variables we chose following El Ghoul et al. (2011): the debt-to-enterprise-value ratio, the market value of equity and the market-to-book ratio. We gather environmental and social performance data from ASSET4, which is one of the world’s leading providers of ESG information. Finally, we obtain data that allow the grouping of stocks using several classifications. Country-specific variables obtained are the legal origin (La Porta et al. 1998), the level of security regulation and the level of disclosure requirements (the last two from Hail and Leuz 2006), the level of earnings management (Leuz et al. 2003), and the interim reporting frequency (DeFond et al. 2007). We further obtain industry classifications based on the impact on stakeholders (Jackson and Apostolakou 2010) and industry profile (Roberts 1992). Finally, we obtain the number of cross-listings for each stock using data from Datastream.

We start from 4047 firms with available data on environmental and social scores over the period 2002–2015. Excluding missing values and firms from countries with less than 15 observations, we end up with a sample of 3822 firms from 52 countries. The US is the most represented country in our dataset, accounting for about 30% of the observations, followed by Japan (12%) and the UK (9%). The most important industries are the financial sector (about 20% of the observations), industrials (19%), and consumer services (13%). Table 10 in the Appendix provides the sample breakdown by country, industry and year. To limit the impact of anomalous values and outliers, we follow Lehn et al. (2007) and winsorize risk measures and control variables: risk measures are winsorized at the 5% level, while control variables are winsorized at the 1% level. Table 1 provides descriptive statistics for the equity risk measures, the CSR measures, and the control and contingency variables. All these variables are described in what follows.

**Table 1:** Descriptive statistics

		N	Mean	SD	Min	Max
*Equity risk measures*						
$\sigma$	Stock price volatility	33,125	0.36	0.15	0.17	0.71
$\beta$	Stock market beta	33,125	0.94	0.46	0.16	1.86
$\beta ^{-}$	Downside beta	33,125	0.97	0.54	0.03	2.04
$\sigma _{\epsilon }$	Idiosyncratic risk	33,125	0.31	0.13	0.15	0.63
*CSR measures*						
$\mathtt {ES}$	Environmental and social score	33,125	0.51	0.29	0.06	0.98
$\mathtt {Env}$	Environmental score	33,125	0.51	0.32	0.08	0.98
$\mathtt {Soc}$	Social score	33,125	0.51	0.31	0.03	0.99
$\mathtt {EmRed}$	Emission reduction	33,125	0.51	0.32	0.07	0.98
$\mathtt {ProdInn}$	Product innovation	33,125	0.49	0.31	0.08	1.00
$\mathtt {ResRed}$	Resource reduction	33,125	0.51	0.32	0.06	0.98
$\mathtt {Comm}$	Community	33,125	0.52	0.31	0.03	0.97
$\mathtt {HumRig}$	Human rights	33,125	0.48	0.31	0.02	1.00
$\mathtt {DivOpp}$	Diversity and opportunity	33,125	0.50	0.31	0.04	0.99
$\mathtt {EmplQual}$	Employment quality	33,125	0.51	0.30	0.03	0.99
$\mathtt {HealSaf}$	Health and safety	33,125	0.49	0.31	0.02	0.99
$\mathtt {TrainDev}$	Training and development	33,125	0.52	0.31	0.05	0.97
$\mathtt {ProdRes}$	Product responsibility	33,125	0.51	0.30	0.02	0.99
*Control variables*						
$\mathtt {D/EV}$	Debt-to-enterprise-value ratio	33,125	27.93	22.84	0.00	90.32
$\ln \mathtt {MV}$	Natural log of equity market value in USD	33,125	14.09	2.33	7.45	21.35
$\mathtt {MTBV}$	Market-to-book ratio	33,125	2.49	1.90	0.54	7.84
*Contingency variables*						
$\mathtt {CommonLaw}$	Common-law country	33,125	0.60	0.49	0.00	1.00
$\mathtt {HighSecReg}$	High security regulation	31,156	0.50	0.50	0.00	1.00
$\mathtt {HighDisReq}$	High disclosure requirement	31,156	0.44	0.50	0.00	1.00
$\mathtt {HighAemScore}$	High earnings management	30,240	0.40	0.49	0.00	1.00
$\mathtt {HighIrFreq}$	High interim reporting frequency	28,489	0.50	0.50	0.00	1.00
$\mathtt {HighImpact}$	High impact on stakeholder industry	33,125	0.22	0.41	0.00	1.00
$\mathtt {HighProfile}$	High profile industry	33,125	0.31	0.46	0.00	1.00
$\mathtt {HighCl}$	High number of cross-listings	28,823	0.59	0.49	0.00	1.00

The table shows the number of observations (N), means (Mean), standard deviations (SD), minima (Min) and maxima (Max) for all the variables in our sample of 3822 firms observed over the period 2002–2015. Risk measures are calculated using daily data. $\sigma$ and $\sigma _{\epsilon }$ figures are annualized

#### Equity risk measures

We use four risk measures as dependent variable in our analysis: stock price volatility ($\sigma$), equity beta ($\beta$), downside risk beta ($\beta ^{-}$), and residual volatility ($\sigma _{\epsilon }$). These variables capture different components of firm risk. We estimate the parameters using Ordinary Least Squares (OLS) regressions of daily stock returns on the market:

1 $$\begin{aligned} r_{i,t}=\alpha _{i}+\beta _{i}r_{m,t}+\epsilon _{i,t} \, , \end{aligned}$$

where $r_{i,t}$ and $r_{m,t}$ are returns from firm *i* and the market *m* measured in USD at time *t*. We use the MSCI World index as the market index. Exposure to systematic risk is given by the equity beta $\beta _{i}$. We measure residual risk $\sigma _{\epsilon ,i}$ by the standard deviation of the idiosyncratic error term $\epsilon _{i,t}$. Total risk $\sigma _{i}$ is given by the standard deviation of stock returns.^3^ To consider the special role of downside risk, we follow Ang et al. (2006) and estimate $\beta ^{-}_{i}$ as the beta of stock *i* to the market, conditional on the market return observed below its mean ${\bar{r}}_{m}$: $\beta ^{-}_{i} = \mathrm {Cov}(r_{i,t},r_{m,t}|r_{m,t}< {\bar{r}}_{m})/\mathrm {Var}(r_{m,t}|r_{m,t} < {\bar{r}}_{m})$.

We estimate $\sigma _{i}$, $\beta _{i}$, $\beta ^{-}_{i}$ and $\sigma _{\epsilon ,i}$ separately for each year a firm is present in our dataset and annualize volatility measures. Across our sample, the average annualized volatility equals 36%, the idiosyncratic risk is about 31%, the average $\beta$ equals 0.94, and the average value of downside $\beta$ is about 0.97.

#### CSR measures

Our variables of interest are CSR indicators. Creating representative measures of CSR performance is a difficult task as CSR is a multidimensional construct and there are many specialized data providers: ASSET4, KLD, RobecoSam, Sustainalytics, and Vigeo Eiris. After excluding KLD as it provides only US data, we have chosen ASSET4 for four main reasons. First, Berg et al. (2020) emphasize that among the major data providers ASSET4 has scores highly correlated in the order of magnitude of 0.6–0.8 with the other raters (RobecoSAM, Sustainalytics, Vigeo-Eiris). On the other hand, KLD exhibits the lowest correlations with all the other data providers. Second, the same authors show that, together with RobecoSAM, ASSET4 is the second dataset in terms of covered countries (60 covered countries versus 170 in Sustainalytics, 15 in Vigeo-Eiris and 1 country—the US—in KLD) and that it covers a comparable number of firms (4025) to Sustainalytics (4551) and KLD (4295), that are the biggest datasets. ASSET4 dominates the other data providers in term of individual indicators: 236 indicators versus 155 by Sustainalytics, 75 by KLD, 74 by RobecoSAM, and 37 by Vigeo-Eiris. Third, data is organized in 4 pillars: (i) economic performance; (ii) environmental performance; (iii) (corporate) governance performance; and (iv) social performance. Each year a firm receives a score for each pillar, benchmarking its performance with the rest of the firms. Fourth, and probably most importantly, ASSET4 considers all firms included in the main stock market indexes, regardless of their policies on CSR communication. This feature of ASSET4 reduces the risk of self-selection bias stemming from self-reporting that characterizes most CSR studies (Shahzad and Sharfman 2017). We provide additional details on our measures and ASSET4 in the Appendix (Sect. A.1).

Similar to Ioannou and Serafeim (2012), we gather environmental and social scores from ASSET4 and exclude the economic and corporate governance dimensions because the latter are less connected with the notion of SRI (Lys et al. 2015). Following Waddock and Graves (1997) and Hillman and Keim (2001), we create an equally-weighted combination of the environmental and social scores provided by ASSET4 to capture CSR performance ($\mathtt {ES}$). In addition, we also consider the constituents of these two pillars separately. Table 1 lists all these constituents and shows that all our CSR scores have an average value of about 0.5.

#### Contingency variables

Specific country characteristics are crucial to understanding the corporate governance choices of firms in different countries, and influence the ability of insiders to divert resources from a company, thus increasing firm risk. La Porta et al. (1998) have shown that the legal origin is an important determinant of the level of investor protection, with common law countries dominating other legal origins. $\mathtt {CommonLaw}$ is our dummy variable indicating firms based in common-law countries (about 60% of our observations). To capture the strength of security regulation and disclosure requirements, we follow Hail and Leuz (2006) and generate two additional dummy variables. $\mathtt {HighSecReg}$ indicates firms incorporated in countries where the level of security regulation is above the median. $\mathtt {HighDisReq}$ equals one for firms based in countries where the level of disclosure requirements is above the median.^4^ To complement the measures of investor protection, we also use a dummy variable indicating firms based in countries where the aggregate earnings management score by Leuz et al. (2003) is above the median ($\mathtt {HighAemScore}$) and a dummy variable indicating firms based in countries where the interim reporting frequency is above the median ($\mathtt {HighIrFreq}$, DeFond et al. 2007).

Based on the industry classification and the definition in Jackson and Apostolakou (2010), we determine if a firm operates in a sector with high impact on stakeholders such as Basic Materials, Oil and Gas, and Utilities ($\mathtt {HighImpact}$). Furthermore, borrowing the method from Roberts (1992) we create a dummy variable ($\mathtt {HighProfile}$) indicating firms belonging to high profile industries such as Basic Materials, Consumer Services, Consumer Goods, and Oil and Gas, i.e. industries characterized by consumer visibility, high level of political risk, and concentrated and intense competition. At firm level, we create a dummy variable indicating firms that are cross-listed in a number of stock markets above the median ($\mathtt {HighCl}$).^5^ Finally, to capture the financial-crisis phase, we divide our sample into three periods: 2002-2006 (pre-crisis), 2007-2009 (during the crisis), and 2010-2015 (post-crisis).

### Empirical model

To formally test the hypotheses outlined in Sect. 2, we use a regression-based approach. Our baseline dynamic regression model is

2 $$\begin{aligned} y_{i,t}=\alpha y_{i,t-1}+\theta \mathtt {CSR}_{i,t-1}+\varvec{\gamma }'\varvec{x}_{i,t-1} +\mu _{i}+\tau _{t}+\varepsilon _{i,t} \,, \end{aligned}$$

where $y_{i,t}$ is a risk measure (either $\sigma$, $\beta$, $\beta ^{-}$ or $\sigma _{\epsilon }$) for firm *i* at time *t*, $\alpha$ and $\theta$ are coefficients, $\varvec{\gamma }$ is a vector of coefficients, $\mathtt {CSR}_{i,t-1}$ is a CSR performance measure (which can be either a composite score or one of its components), $\varvec{x}_{i,t-1}$ is a vector of control variables, $\mu _{i}$ denotes an unobservable time-constant firm effect, $\tau _{t}$ indicates an unobservable firm-constant time effect and $\varepsilon _{i,t}$ indicates a zero-mean idiosyncratic stochastic error term.

For the estimation of Eq. (2), we use the two-step Arellano and Bover (1995)/Blundell and Bond (1998) GMM system estimator (Baltagi 2013). We base inference on Windmeijer (2005) robust standard errors. In the CSR literature, a common concern is endogeneity stemming from omitted variables and/or reverse causality. While pooled-OLS and fixed-effects estimators are commonly used in empirical corporate finance studies, they are biased in case of autoregressive effects and endogeneity. On the one hand, our GMM system approach can account for unobservable heterogeneity (stemming from omitted time-invariant effects) similar to the fixed-effects estimator. On the other hand, this estimator has the advantage over the fixed-effects estimator of handling autoregressive memory in risk measures and possible endogeneity of CSR due to reverse causality (García-Herrero et al. 2009; Bontempi and Golinelli 2012; Wintoki et al. 2012; Ellul and Yerramilli 2013). For example, Ellul and Yerramilli (2013) use a dynamic panel GMM estimator to alleviate the concern that the relationship between a risk management index and tail risk of the US banks is dynamically endogenous. In Eq. (2), the coefficient $\theta$ is the one of main interest, as it measures the marginal effect of CSR on risk, $\partial y_{i,t} / \partial \mathtt {CSR}_{i,t-1}$. Since we estimate dynamic models, $\theta$ can be interpreted as a short-run effect (Greene 2011). The corresponding long-run effect, $\lambda$, is given by $\lambda = 1/(1-\alpha )\theta$.

When investigating the role of contingency variables, we estimate models for each subgroup (e.g., common-law vs. civil-law countries). This is equivalent to leaving all coefficients free to vary between subgroups (similar to the approach used in Ghosh and Tang 2015) and estimating fully interacted models.

## Empirical analysis

### Baseline results

Estimation results of the baseline model, presented in Table 2, confirm the negative relationship between CSR and risk we expected based on previous literature (Heinkel et al. 2001; Renneboog et al. 2011). The expected relationship is statistically significant: A higher environmental and social score leads to lower total risk ($\sigma$), systematic risk ($\beta$), and idiosyncratic risk ($\sigma _{\epsilon }$). The coefficient for downside risk ($\beta ^{-}$) has the expected negative sign but is not statistically significant. The overall finding of a negative effect of CSR on risk measures is consistent with the literature (e.g., Jo and Na 2012). To illustrate the magnitude of the effects, we consider first systematic risk. An increase of one standard deviation in $\mathtt {ES}$ is followed by a decrease of 0.07 standard deviations of $\beta$ in the subsequent year. The value of -0.07 is obtained as the product of the coefficient in Table 2 with the ratio of the standard deviations of $\mathtt {ES}$ and $\beta$ given in Table 1, i.e. ($-0.1109 \cdot 0.29 / 0.46$). The economic significance is best seen from the long-term effect. Based on the value of the autoregressive coefficient of 0.2943, the long-term marginal effects of CSR on beta is 1.4 times larger than the short-term effect ($1.42 = 1 / (1-0.2943)$). The resulting standardized long-term reduction in $\beta$ is thus about 10%. For total risk, this figure is about 5%^6^ and for idiosyncratic risk about 7%.

In the last three columns of Table 2, we consider three alternative risk measures for the relationship between CSR and risk: $\beta ^{\mathtt {MKT}}$, $\beta ^{\mathtt {SML}}$ and $\beta ^{\mathtt {HML}}$. We obtain these three risk measures by estimating the Fama-French three-factor model (Fama and French 1993) on the sample of returns. The idea of this robustness check is to obtain an alternative measure of systematic risk ($\beta ^{\mathtt {MKT}}$), after controlling for the size and book-to-market factors. The results for $\beta ^{\mathtt {MKT}}$ are significant and even slightly more pronounced than those for $\beta$. We do not have any strong a priori expectation for the relationship between CSR and $\beta ^{\mathtt {SML}}$ or $\beta ^{\mathtt {HML}}$. Our empirical analysis shows that CSR strategies affect the sensitivity of firms to market risk, but there is no statistically significant relationship to the size and book-to-market factors.

**Table 2:** Baseline models

	$\sigma$	$\beta$	$\beta ^{-}$	$\sigma _{\epsilon }$	$\beta ^{\mathtt {MKT}}$	$\beta ^{\mathtt {SMB}}$	$\beta ^{\mathtt {HML}}$
$$\mathtt {ES}_{t - 1}$$	− 0.0139$$^{**}$$	− 0.1109$$^{***}$$	− 0.0662	− 0.0184$$^{***}$$	− 0.1496$$^{***}$$	− 0.0058	− 0.0082
	[0.0066]	[0.0349]	[0.0465]	[0.0060]	[0.0322]	[0.0577]	[0.0699]
$$\sigma _{t-1}$$	0.4075$$^{***}$$						
	[0.0161]						
$$\beta _{t-1}$$		0.2943$$^{***}$$					
		[0.0158]					
$$\beta ^{-}_{t-1}$$			0.0679$$^{***}$$				
			[0.0129]				
$$\sigma _{\epsilon ,t-1}$$				0.4037$$^{***}$$			
				[0.0170]			
$$\beta ^{\mathtt {MKT}}_{t-1}$$					0.3271$$^{***}$$		
					[0.0144]		
$$\beta ^{\mathtt {SMB}}_{t-1}$$						0.1197$$^{***}$$	
						[0.0170]	
$$\beta ^{\mathtt {HML}}_{t-1}$$							0.1862$$^{***}$$
							[0.0132]
$$\mathtt {D/EV}_{t - 1}$$	0.0009$$^{***}$$	− 0.0005	0.0005	0.0009$$^{***}$$	− 0.0009$$^{**}$$	0.0020$$^{**}$$	0.0090$$^{***}$$
	[0.0001]	[0.0005]	[0.0006]	[0.0001]	[0.0004]	[0.0010]	[0.0010]
$$\ln \mathtt {MV}_{t - 1}$$	− 0.0010	− 0.0726$$^{***}$$	− 0.0858$$^{***}$$	0.0007	− 0.0590$$^{***}$$	0.1541$$^{***}$$	− 0.0383$$^{***}$$
	[0.0014]	[0.0059]	[0.0086]	[0.0012]	[0.0047]	[0.0177]	[0.0133]
$$\mathtt {MTBV}_{t - 1}$$	0.0062$$^{***}$$	0.0173$$^{***}$$	0.0179$$^{***}$$	0.0049$$^{***}$$	0.0101$$^{***}$$	− 0.0149$$^{**}$$	− 0.0547$$^{***}$$
	[0.0008]	[0.0035]	[0.0045]	[0.0007]	[0.0036]	[0.0071]	[0.0090]
constant	0.1256$$^{***}$$	1.6795$$^{***}$$	2.1432$$^{***}$$	0.1026$$^{***}$$	1.5431$$^{***}$$	− 1.6399$$^{***}$$	0.6511$$^{***}$$
	[0.0219]	[0.0896]	[0.1290]	[0.0181]	[0.0725]	[0.2466]	[0.1964]
Ar.-Bond	− 1.24	12.19***	7.58***	0.73	3.54***	10.01***	− 3.03
Sargan	787.94***	1142.54***	983.50	662.50***	879.92***	799.12***	710.46***
year effects	yes	yes	yes	yes	yes	yes	yes
N	29238	29238	29238	29238	29230	29230	29230

*p<0.10, **p<0.05, ***p<0.01

This table shows estimates of the model in Eq. (2) for all equity risk measures. Estimates are based on the two-step Arellano and Bover (1995)/Blundell and Bond (1998) GMM system estimator. Inference is based on Windmeijer (2005) robust standard errors, reported in square brackets

### Heterogeneity in the CSR-risk channel

After having established the general effect of CSR on risk, we investigate how the strength of the relationship varies across different environments and company characteristics. As outlined in hypotheses H1, H2, and H3, the benefits and costs of CSR are likely to depend on country, industry and firm characteristics and might be time-varying.

In Table 3, we consider the moderating role of the legal and financial information environments on the relationship between CSR and risk (H1). Firms are divided into subgroups according to their country characteristics. Panel A focuses on the common/civil-law split, on the level of security regulation, and on the level of disclosure requirements. While the point estimates are consistent across the variables related to the legal environment, we find statistical significance based on difference tests only for the common/civil law split.^7^ We find that in countries where shareholders are less protected, CSR plays a stronger role in mitigating risk. The differences are most pronounced for idiosyncratic risk, and sizable for volatility and downside risk. The point estimates indicate that the marginal effect of CSR on idiosyncratic risk is about 13 times greater in civil law countries than in (shareholder friendly) common law countries. In Panel B, we focus on the financial information environment. Results resemble those in Panel A: Again the risk reduction effect is more pronounced for countries with weaker investor protection characteristics. We find statistically significant differences for both country characteristics analyzed, the level of earnings management and the reporting frequency. For countries where earnings management is more widespread, CSR plays a stronger role in mitigating risk for all risk measures analyzed. Our results are similar for the split on the interim reporting frequency, albeit more pronounced for $\beta$ and $\beta ^{-}$, and somewhat weaker for $\sigma$ and $\sigma _{\epsilon }$. The results in Panels A and B are consistent with previous studies that show that the strength of beneficial effects through CSR activities depends on country characteristics. For example, Dhaliwal et al. (2014) find that the relationship between CSR disclosure and the cost of equity capital is stronger in countries with higher levels of financial opaqueness, and Krueger et al. (2019) find that the protection of investor reputation, their ethical considerations, and their legal and fiduciary duties are strong investment motives for institutional investors. Our findings provide strong evidence that the benefits from CSR – in terms of reduced equity risk – are stronger for countries with weaker fundamentals. For companies from these countries, investors benefit from lower volatility subsequent to an increase in ESG scores, while the lower systematic risk translates into a lower cost of capital for companies.

In Table 4, we report our findings on hypothesis H2, which questions whether the effect of CSR on risk is contingent on industry and company characteristics. First, following Jackson and Apostolakou (2010), firms are divided according to whether they belong to industries with a high or low impact on stakeholders. Then, along the line of Roberts (1992), we split firms into high and low profile industries. Consistent with our hypothesis H2, we find differences in the effect of CSR on risk due to industry characteristics. Risk mitigation tends to be stronger for industries with a high impact on stakeholders or a high profile, that are often referred to as controversial industries. The economically and statistically most significant results relate to $\sigma$ and $\sigma _{\epsilon }$. As for the latter, estimated coefficients are 6 times larger in high impact industries and 3 times larger in high profile industries. Table 4 further analyzes the possible importance of cross-listings on the relationship between CSR and risk. The cross-listing literature finds that cross-listing firms comply with stricter foreign standards (e.g., Pagano et al. 2002), so we expect a lower marginal benefit from additional risk mitigation via CSR activities for these firms. The strikingly different point estimates are consistent with this view, but imprecisely estimated; the differences are not statistically significant.^8^ In summary, the analysis of the moderating role of industry and firm characteristics in Table 4 reinforces the finding that it is the firms for which CSR matters most who reap the highest benefits.

**Table 3:** Country-level contingency analysis

	$\mathtt {CommonLaw}$	$\mathtt {CivilLaw}$	$\Delta$	$\mathtt {HighSecReg}$	$\mathtt {LowSecReg}$	$\Delta$	$\mathtt {HighDisReq}$	$\mathtt {LowDisReq}$	$\Delta$
*Panel A-Legal environment*									
$\sigma$	0.0009	− 0.0233**	0.0242*	0.0007	− 0.0152	0.0159	− 0.0051	− 0.0255***	0.0204
	[0.0087]	[0.0098]	[0.0131]	[0.0106]	[0.0092]	[0.0140]	[0.0112]	[0.0084]	[0.0140]
$\beta$	− 0.0896**	− 0.0746	− 0.0150	− 0.0076	− 0.0718	0.0642	0.0108	− 0.0039	0.0147
	[0.0394]	[0.0526]	[0.0657]	[0.0445]	[0.0509]	[0.0676]	[0.0472]	[0.0477]	[0.0671]
$\beta ^{-}$	− 0.0293	− 0.1312*	0.1019	− 0.0302	− 0.1643**	0.1341	− 0.0146	− 0.1161**	0.1015
	[0.0506]	[0.0676]	[0.0844]	[0.0523]	[0.0642]	[0.0828]	[0.0575]	[0.0585]	[0.0820]
$\sigma _{\epsilon }$	− 0.0025	− 0.0343***	0.0318***	− 0.0079	− 0.0215***	0.0136	− 0.0096	− 0.0280***	0.0184
	[0.0080]	[0.0086]	[0.0117]	[0.0095]	[0.0082]	[0.0125]	[0.0099]	[0.0077]	[0.0125]

	$\mathtt {LowAemScore}$	$\mathtt {HighAemScore}$	$\Delta$	$\mathtt {HighIrFreq}$	$\mathtt {LowIrFreq}$	$\Delta$			
*Panel B-Financial information environment*									
$\sigma$	0.0122	− 0.0288***	0.0410***	0.0106	− 0.0142	0.0248			
	[0.0092]	[0.0104]	[0.0139]	[0.0111]	[0.0103]	[0.0151]			
$\beta$	− 0.0814*	− 0.1128**	0.0314	− 0.0135	− 0.1473**	0.1338*			
	[0.0420]	[0.0565]	[0.0704]	[0.0457]	[0.0585]	[0.0742]			
$\beta ^{-}$	− 0.0536	− 0.2084***	0.1548*	− 0.0470	− 0.1931***	0.1461			
	[0.0517]	[0.0674]	[0.0849]	[0.0536]	[0.0711]	[0.0890]			
$\sigma _{\epsilon }$	0.0133	− 0.0343***	0.0476***	0.0050	− 0.0173*	0.0223*			
	[0.0085]	[0.0092]	[0.0125]	[0.0100]	[0.0089]	[0.0134]			

*p<0.10, **p<0.05, ***p<0.01

The table shows results concerning the moderating role of the legal (Panel A) and financial information (Panel B) environments on the relationship between CSR and equity risk. Firms are divided into subgroups according to their country characteristics. Panel A focuses on the common/civil-law divide, on the level of security regulation and on the level of disclosure requirements. Panel B focuses on the level of earnings management and the interim reporting frequency. Only the coefficient $\theta$ of each model (one for each equity risk measure and country-level division) is reported in the table together with the subgroup difference test ($\Delta$). Estimates are based on the two-step Arellano and Bover (1995)/Blundell and Bond (1998) GMM system estimator. Inference is based on Windmeijer (2005) robust standard errors, reported in square brackets

**Table 4:** Industry and firm level contingency analysis

	$\mathtt {HighImpact}$	$\mathtt {LowImpact}$	$\Delta$	$\mathtt {HighProfile}$	$\mathtt {LowProfile}$	$\Delta$	$\mathtt {HighCl}$	$\mathtt {LowCl}$	$\Delta$
$\sigma$	− 0.0511***	0.0019	− 0.0530***	− 0.0332***	− 0.0101	− 0.0231*	0.0010	− 0.0180**	0.0190
	[0.0157]	[0.0072]	[0.0173]	[0.0109]	[0.0082]	[0.0136]	[0.0149]	[0.0079]	[0.0169]
$\beta$	− 0.0974	− 0.0971**	− 0.0003	− 0.2114***	− 0.0816*	− 0.1298*	− 0.0739	− 0.0939**	0.0200
	[0.0752]	[0.0392]	[0.0848]	[0.0587]	[0.0438]	[0.0732]	[0.0930]	[0.0427]	[0.1023]
$\beta ^{-}$	− 0.0142	− 0.0425	0.0283	− 0.1240	− 0.0412	− 0.0828	0.0193	− 0.1317**	0.1510
	[0.1012]	[0.0534]	[0.1144]	[0.0759]	[0.0587]	[0.0960]	[0.1180]	[0.0567]	[0.1309]
$\sigma _{\epsilon }$	− 0.0550***	− 0.0100	− 0.0450***	− 0.0357***	− 0.0135*	− 0.0222*	− 0.0056	− 0.0261***	0.0205
	[0.0147]	[0.0064]	[0.0160]	[0.0100]	[0.0074]	[0.0124]	[0.0130]	[0.0071]	[0.0148]

*p<0.10, **p<0.05, ***p<0.01

The table shows results concerning the moderating role of industry and firm characteristics on the relationship between CSR and equity risk. Firms are divided according to whether they belong to industries with a high or low impact on stakeholders, industries with a high or low profile, and firms with a number of cross-listings above or below the median. Only the coefficient $\theta$ of each model (one for each equity risk measure and industry-level o firm-level division) is reported in the table together with the subgroup difference test ($\Delta$). Estimates are based on the two-step Arellano and Bover (1995)/Blundell and Bond (1998) GMM system estimator. Inference is based on Windmeijer (2005) robust standard errors, reported in square brackets

In Table 5, we present results concerning hypothesis H3 about changes in the relationship between CSR and risk due to the financial crisis. Before the financial crisis (2002–2006), CSR seems to have had no effect on risk. During the global financial crisis (2007–2009), some risk measures were influenced by CSR. After the financial crisis (2010–2015), all our risk measures were strongly influenced by CSR.^9^ This result is consistent with greater attention to CSR as an instrument to reduce risk elicited by the financial crisis. For example, Guiso et al. (2008) emphasize that investing in stocks requires both a risk-return analysis based on existing data and an ‘act of faith’ that this data is actually reliable. During a sudden decline in the general level of trust, investors are likely to be more worried about the reliability of the financial information they use for their investment decisions. Hence, they need scores such as social capital ratings describing a firm’s values and integrity, and give a valuation premium to the firms that seem more trustworthy.

**Table 5:** Contingency analysis by time

	2002–2006	2007–2009	2010–2015
$\sigma$	− 0.0028	0.0159	− 0.0448***
	[0.0113]	[0.0122]	[0.0119]
$\beta$	− 0.0515	− 0.2331***	− 0.0776**
	[0.0852]	[0.0534]	[0.0360]
$\beta ^{-}$	0.1285	− 0.2864***	− 0.1023**
	[0.1301]	[0.0621]	[0.0485]
$\sigma _{\epsilon }$	− 0.002	0.0031	− 0.0779***
	[0.0103]	[0.0106]	[0.0102]

*p<0.10, **p<0.05, ***p<0.01

The table shows results concerning the moderating role of the financial-crisis phase on the relationship between CSR and equity risk. Observations are divided according to whether they pertain to the pre-, during- or post-crisis periods (i.e., 2002–2006, 2007–2009, 2010–2015). Only the coefficient $\theta$ of each model (one for each equity risk measure and period) is reported in the table. Estimates are based on the two-step Arellano and Bover (1995)/Blundell and Bond (1998) GMM system estimator. Inference is based on Windmeijer (2005) robust standard errors, reported in square brackets

### Robustness

To check for robustness of our results, we investigate in this section subdimensions of ESG, employ fixed-effects estimation as an alternative empirical technique, discuss potential effects of sample composition, and investigate the effect from sustainable index inclusion and exclusion. We further discuss potential effects of heterogeneity in ESG scores across different providers.

*Subdimensions of ESG:* In Table 2, we have presented results on the overall CSR score. It is not clear if the effect from risk reduction is broadly driven by the components of the overall score or mainly present for a specific subdimension. In Table 6, we report results related to its two components. For the sake of brevity, also in Table 6 we report only the coefficient of interest, i.e., $\theta$. Panel A presents the analysis for the environmental score and its subdimensions. The environmental score has a risk-reducing effect, as shown by the negative coefficients in the regressions explaining $\sigma$, $\beta$ and $\sigma _{\epsilon }$. The major role seems to be played by emission reduction ($\mathtt {EmRed}$). This result is consistent with findings showing a positive relationship between emission reduction and firm performance (e.g., Hart and Ahuja 1996). A negative relationship between emission reduction and systematic risk implies a positive relationship between emission reduction and firm value; ‘it does indeed pay to be green’. For the subdimensions product innovation ($\mathtt {ProdInn}$) and resource reduction ($\mathtt {ResRed}$), all signs are negative but statistical significance is weaker. In Panel B we show results for the social score and its subdimensions. The social score has a statistically significant negative effect on $\beta$ and $\sigma _{\epsilon }$. The major role seems to be played by diversity and opportunity ($\mathtt {DivOpp}$). This is consistent with evidence that the promotion of diversity issues and equal opportunities plays an important role for employee satisfaction and increases risk-adjusted shareholder returns; in an international context this effect depends on the extent of labor market rigidity (Edmans 2011; Edmans et al. 2014).

**Table 6:** Environmental and social subdimensions

Variables	$\sigma$	$\beta$	$\beta ^{-}$	$\sigma _{\epsilon }$
*Panel A. Environmental subdimensions*				
$\mathtt {Env}_{t - 1}$	− 0.0114**	− 0.0804***	− 0.0611	− 0.0165***
	[0.0054]	[0.0294]	[0.0387]	[0.0050]
$\mathtt {EmRed}_{t - 1}$	− 0.0092*	− 0.0803***	− 0.0738*	− 0.0109**
	[0.0052]	[0.0283]	[0.0380]	[0.0047]
$\mathtt {ProdInn}_{t - 1}$	− 0.0053	− 0.0138	− 0.0129	− 0.0084**
	[0.0046]	[0.0235]	[0.0315]	[0.0041]
$\mathtt {ResRed}_{t - 1}$	− 0.0057	− 0.0702***	− 0.0449	− 0.0118***
	[0.0047]	[0.0256]	[0.0340]	[0.0043]
*Panel B. Social subdimensions*				
$\mathtt {Soc}_{t - 1}$	− 0.0088	− 0.0805***	− 0.0322	− 0.0105**
	[0.0058]	[0.0304]	[0.0416]	[0.0052]
$\mathtt {Comm}_{t - 1}$	− 0.0047	− 0.0501**	− 0.0297	− 0.0033
	[0.0041]	[0.0198]	[0.0266]	[0.0036]
$\mathtt {HumRig}_{t - 1}$	− 0.0074*	− 0.0516**	− 0.0192	− 0.0066
	[0.0045]	[0.0255]	[0.0345]	[0.0041]
$\mathtt {DivOpp}_{t - 1}$	− 0.0082*	− 0.0499**	− 0.0213	− 0.0088**
	[0.0044]	[0.0246]	[0.0345]	[0.0040]
$\mathtt {EmplQual}_{t - 1}$	− 0.0019	0.0042	0.0165	− 0.0034
	[0.0040]	[0.0180]	[0.0245]	[0.0035]
$\mathtt {HealSaf}_{t - 1}$	0.0003	− 0.0241	− 0.0366	− 0.001
	[0.0047]	[0.0238]	[0.0320]	[0.0040]
$\mathtt {TrainDev}_{t - 1}$	− 0.0053	− 0.0534**	− 0.0223	− 0.0063
	[0.0045]	[0.0241]	[0.0325]	[0.0041]
$\mathtt {ProdRes}_{t - 1}$	0.0023	− 0.0187	− 0.0003	− 0.0015
	[0.0040]	[0.0206]	[0.0278]	[0.0036]

*p<0.10, **p<0.05, ***p<0.01

Estimates of the effect of all environmental (Panel A) and social (Panel B) subdimensions of CSR on equity risk. Each panel presents the effect of a specific subdimension. Four models are presented in each panel (one model for each risk measure). Only the coefficient $\theta$ of each model is reported in the table. Estimates are based on the two-step Arellano and Bover (1995)/Blundell and Bond (1998) GMM system estimator. Inference is based on Windmeijer (2005) robust standard errors, reported in square brackets

*Estimation method * A possible concern with our analysis is related to the potential endogeneity of the CSR-risk relationship. More specifically, as already mentioned, the relationship between CSR and risk could be bidirectional (e.g., the riskiest companies could decide to invest in CSR to reduce their risk), causing a problem of reverse causality. This effect would be even more serious if we consider the simultaneous effect of CSR on risk (rather than a lagged effect as in Table 2). To take these potential problems into account, we estimate additional models where CSR is treated as an endogenous variable and can simultaneously affect risk measures. Results are reported in Table 7 (Panel A). While the coefficient for $\beta$ is no longer statistically significant in this specification, the results for the other risk measures carry over from Table 2.

A somewhat related concern about the GMM estimator is whether the two underlying specification assumptions of serial uncorrelation and validity of instruments are met with the data. We test our models using the Arellano and Bond (1991) test for serial correlation in the first-differenced errors and the Sargan (1958) test for over-identifying restrictions. The Arellano-Bond tests do not reject most of our models (e.g., for the first model in Table 2 the p-value is much greater than 10%), and when it does so, augmenting the model specification with additional lags solves the problem without altering the qualitative nature of the results. So this test is not a real concern. The Sargan tests of over-identifying restrictions, however, do reject the null hypothesis in most of the cases. While suggesting some caution in interpreting our results, these rejections might also be due to heteroscedasticity in the data, since the Sargan test tends to be unreliable in presence of heteroscedasticity (Arellano and Bond 1991). Heteroscedasticity is indeed likely to characterize our heterogeneous dataset of firms and that motivates our choice of using Windmeijer’s finite-sample correction for the standard errors (Windmeijer 2005).^10^

To alleviate concerns about the dynamic panel regression specification, we estimate the models of Table 2 using the fixed-effects estimator as an additional robustness check. We report the results in Panel B of Table 7. The risk-mitigating effect of higher ESG ratings on subsequent risk measures is highly significant and resembles the results of Table 2. Furthermore, the results of all other models in the paper basically carry over when we use the fixed-effects estimator. In most regressions the effect of CSR is even more pronounced than in our base estimations. We provide the details in the Appendix in Tables 11 (country characteristics), 12 (industry characteristics), and 13 (time periods). This is a somewhat expected result. The GMM estimator allows taking into account autoregressive memory and endogeneity but is more subject to sample variability. Fixed-effects models, while less flexible, are less demanding on the data.

*Sample composition* A further concern with our empirical analysis is a potential bias of our database towards a limited set of countries or industries. US and financial firms are the most represented in our sample, accounting for about 30% and 20% of the observations, respectively. However, it is worth noting that while they are the most represented, they are not over-represented as world economic statistics suggest. For example, the 16th annual Forbes Global 2000 list, including listed companies from 60 countries, shows that US firms represent almost 30% of the total. Nevertheless, to take this potential problem into account, we run regression models excluding first US firms and then financial firms. Results reported in Table 7 (Panels C and D) seem qualitatively unaffected, suggesting they can withstand these sample selection changes.

**Table 7:** Robustness checks

Variables	$\sigma$	$\beta$	$\beta ^{-}$	$\sigma _{\epsilon }$
*Panel A-Endogeneity and contemporaneous effect of *$\mathtt {ES}$				
$\mathtt {ES}_{t}+\mathtt {ES}_{t - 1}$	− 0.0508***	− 0.0306	0.0618	− 0.0623***
	[0.0103]	[0.0573]	[0.0779]	[0.0096]
*Panel B-Fixed effects*				
$\mathtt {ES}_{t - 1}$	− 0.0093*	− 0.0769***	− 0.0734***	− 0.0092**
	[0.0051]	[0.0211]	[0.0249]	[0.0046]
*Panel C-Non-US firms*				
$\mathtt {ES}_{t - 1}$	− 0.0333***	− 0.0354	− 0.1111**	− 0.0388***
	[0.0076]	[0.0413]	[0.0507]	[0.0070]
*Panel D-Non-financial firms*				
$\mathtt {ES}_{t - 1}$	− 0.0190***	− 0.1119***	− 0.0554	− 0.0227***
	[0.0070]	[0.0381]	[0.0496]	[0.0065]

*p<0.10, **p<0.05, ***p<0.01

This table provides some robustness checks. In Panel A, CSR is treated as an endogenous variable and can simultaneously affect equity risk measures. Reported figures are the sum of contemporaneous and lagged effect of $\mathtt {ES}$. In Panel B, we report estimates using a static fixed-effects estimator. In Panels C and D, we exclude US and financial firms from the sample, respectively. With the exception of Panel B, estimates are based on the two-step Arellano and Bover (1995)/Blundell and Bond (1998) GMM system estimator. Inference is based on Windmeijer (2005) robust standard errors, reported in square brackets

*Index inclusion* As an additional test, we consider the moderating effect of the inclusion and exclusion from the Dow Jones Sustainability World Index (DJSWI). According to the so-called Information Hypothesis (Harris and Raviv 1985), the revision in the composition of a stock market index can be considered as new information able to impact the expected value of a firm and its risk profile (Harris and Gurel 1986). More specifically, the inclusion into a sustainability index is typically good news as the involved firm becomes a member of an exclusive group of firms characterized by a certified status of CSR excellence (Lamoureux and Wansley 1987). In Table 8, we consider this inclusion/exclusion effect through two dummy variables, $\mathtt {InDJ}$ and $\mathtt {OutDJ}$ respectively, and their interactions with $\mathtt {ES}$. Even though this analysis is available only for the years following 2005 due to data availability, our results substantially confirm our baseline models: CSR and risk are negatively related. This negative relationship is significant in all models. All in all, the effect of $\mathtt {InDJ}$ and $\mathtt {OutDJ}$ appears modest, suggesting that our considerations based on baseline models are robust to this specification change.

**Table 8:** Inclusion in and exclusion from the Dow Jones Sustainability World Index

Variables	$\sigma$	$\beta$	$\beta ^{-}$	$\sigma _{\epsilon }$
$\mathtt {ES}_{t - 1}$	− 0.0131*	− 0.1246***	− 0.2003***	− 0.0202***
	[0.0077]	[0.0343]	[0.0441]	[0.0071]
$\mathtt {InDJ}_{t - 1}$	0.0307	0.1420	0.1559	0.0317
	[0.0345]	[0.1261]	[0.1750]	[0.0300]
$\mathtt {InDJ}_{t - 1} \times \mathtt {ES}_{t - 1}$	− 0.0279	− 0.1770	− 0.1627	− 0.0292
	[0.0407]	[0.1448]	[0.2009]	[0.0354]
$\mathtt {OutDJ}_{t - 1}$	0.0405	0.1310	0.2453	0.0573
	[0.0399]	[0.1356]	[0.2566]	[0.0400]
$\mathtt {OutDJ}_{t - 1} \times \mathtt {ES}_{t - 1}$	− 0.041	− 0.1429	− 0.2734	− 0.0677
	[0.0469]	[0.1634]	[0.3018]	[0.0464]
$\sigma _{t-1}$	0.3922***			
	[0.0170]			
$\beta _{t-1}$		0.2979***		
		[0.0166]		
$\beta ^{-}_{t-1}$			0.1270***	
			[0.0140]	
$\sigma _{\epsilon ,t-1}$				0.3892***
				[0.0182]
$\mathtt {D/EV}_{t - 1}$	0.0010***	− 0.0001	0.0005	0.0010***
	[0.0001]	[0.0005]	[0.0006]	[0.0001]
$\ln \mathtt {MV}_{t - 1}$	− 0.0046***	− 0.0944***	− 0.1083***	− 0.0021
	[0.0016]	[0.0060]	[0.0085]	[0.0013]
$\mathtt {MTBV}_{t - 1}$	0.0062***	0.0154***	0.0147***	0.0045***
	[0.0009]	[0.0037]	[0.0047]	[0.0008]
constant	0.2432***	1.9789***	2.3565***	0.1947***
	[0.0248]	[0.0931]	[0.1274]	[0.0199]
year effects	yes	yes	yes	yes
N	24216	24216	24216	24216

*p<0.10, **p<0.05, ***p<0.01

This table shows the moderating effect of the inclusion in and exclusion from the Dow Jones Sustainability World Index on the relationship between CSR and equity risk. Estimates are based on the two-step Arellano and Bover (1995)/Blundell and Bond (1998) GMM system estimator. Inference is based on Windmeijer (2005) robust standard errors, reported in square brackets

*Heterogeneity of ESG scores* Berg et al. (2020) find that the correlation of ESG scores across data providers is astonishingly low. ESG ratings of ASSET4—which we use in our study—have relatively high correlations in the order of magnitude of 0.6–0.8 with those of a subgroup of other well-known rating providers such as RobecoSAM, Sustainalytics, and Vigeo-Eiris but low correlation with those by KLD (which, however, covers US firms only). While we cannot rule out that our results are specific to the choice of the ASSET4 database, we gather ESG ratings from Sustainalytics (via Bloomberg) for the years 2014-2015. While the data do not allow us to run dynamic models, we find for the subsample investigated a slightly higher correlation between ASSET4 and Sustainalytics ESG scores (0.73 in our data versus 0.67 in Berg et al. (2020)). While it would be interesting to formally test the validity of our results across data providers, a change in the rating methodology of ASSET4 allows to shed further light on the robustness of our results. We discuss this evidence in Sect. 4.4.

### Recent evidence

ASSET4 has recently changed the methodology behind its ESG measures, providing both the original and the new versions for the year 2016 and the new scores since then. With the new methodology, a company’s score is determined based on its relative performance in comparison to peers within the same industry group or country. This change makes it difficult to simply extend our sample into the more recent period, and is likely to hamper cross-country and cross-industry analysis. Yet it allows to test the robustness of our main empirical results in a separate analysis. In addition, the more recent period potentially allows to gain insights from the impact of the COVID-19 crisis on the CSR-risk relationship. We thus collect ESG scores based on the new Refinitiv methodology for the years 2016-2020 and the companies in our original sample.

To allow for a specific effect of the COVID-19 crisis, we modify the baseline regression from equation (2) and add an interaction term $\mathtt{CSR_{i,t-1} \times d_{2020}}$, where $\mathtt{CSR_{i,t-1}}$ is the score of a specific ESG subdimension and $d_{2020}$ is a calendar year 2020 dummy variable. We thus estimate Eq. (3). We report the coefficients $\theta$ and $\phi$ for the combined environmental and social scores (ES) and for the subdimensions environmental (Env), and social (Soc) separately in Table 9.

3 $$\begin{aligned} y_{i,t}=\alpha y_{i,t-1}+\theta \mathtt {CSR}_{i,t-1} +\phi \left( \mathtt{CSR_{i,t-1} \times d_{2020}}\right) + \lambda \mathtt{d_{2020}} +\varvec{\gamma }'\varvec{x}_{i,t-1} +\mu _{i}+\varepsilon _{i,t} \end{aligned}$$

**Table 9:** CSR effects on risk measures from 2016–2020

Variables	$\sigma$	$\beta$	$\beta ^{-}$	$\sigma _{\epsilon }$
$\mathtt {ES}_{t - 1}$	− 0.1386***	− 0.1074	− 0.2204**	− 0.1002***
$\mathtt {ES}_{t - 1} \times d_{2020}$	0.0167	− 0.1418***	− 0.1298*	0.0043
$\mathtt {Env}_{t - 1}$	− 0.0959***	− 0,0887	− 0.2292***	− 0.0705***
$\mathtt {Env}_{t - 1} \times d_{2020}$	− 0,0146	− 0.1195***	− 0,0691	− 0,0108
$\mathtt {Soc}_{t - 1}$	− 0.1186***	− 0,0793	− 0,0878	− 0.0826***
$\mathtt {Soc}_{t - 1} \times d_{2020}$	0.0572***	− 0.1176**	− 0.1709**	0.0252*

This table shows estimates of the model in equation 3 for the equity risk measures total risk ($\sigma$), beta ($\beta$), downside beta ($\beta ^{-}$), and idiosyncratic risk ($\sigma _{\epsilon }$). We report the coefficients on the lagged CSR measure (based on the new Refinitiv methodology) of $\mathtt {CSR}_{i,t-1}$ and its interaction effect with a 2020 calendar year dummy, $\mathtt{CSR_{i,t-1} \times d_{2020}}$. We run separate regressions for the CSR measures $\mathtt{ES}$, $\mathtt{Env}$, and $\mathtt{Soc}$. Estimates are based on the two-step Arellano and Bover (1995)/Blundell and Bond (1998) GMM system estimator. Inference is based on Windmeijer (2005) robust standard errors, 10%, 5%, and 1% significance levels are indicated by ***, **, and *, respectively

Table 9 confirms that higher CSR scores—of all subdimensions—have a moderating effect on important risk measures. During the COVID-19 crisis, the effects tend to be more pronounced. This is shown by the interaction effects that are negative for most subdimensions and risk measures. The largest point estimates can be seen on the downside risk beta for the combined environmental and social score: The direct effect is measured with − 0.22, with a further − 0.13 in 2020. While the direct effect is statistically and economically significant for total risk and idiosyncratic risk with reductions between 0.10 and 0.14, there is no additional effect in 2020.^11^ Interestingly, in the crisis year 2020 our analysis shows a stronger risk reducing effect (indicated by negative coefficients on the cross-terms) for the measures associated with the cost of capital: systematic risk and downside risk. Overall, the risk-mitigating effect of higher ESG scores carries over into the more recent period, with a tendency to even stronger effects during 2020.

## Conclusion

Recently, SRI has drawn a growing interest among academics, corporate managers, and investors. Several authors identify more than one argument to explain why firms implement CSR strategies and investors consider ESG criteria in their investment decisions: opportunism, reputation, and risk reduction. If we focus on the main argument, operational and financial motives suggest a negative effect of CSR on risk. In this article, we investigate this prediction for a global sample of listed firms from 52 countries in the period 2002–2020.

Our empirical analysis clearly shows that CSR reduces risk. Emission reduction and board diversity seem to play a major role in this risk-reduction mechanism. Furthermore, the effect of CSR on risk is contingent on country-, industry- and firm-level characteristics. It is more pronounced in a weaker institutional environment. More specifically, the effect is stronger in civil-law countries, in countries with low security regulation or disclosure requirement levels and where financial information is less widespread. Firms in industries with a high impact on stakeholders or in high profile industries have more advantages to implement CSR strategies than firms in other industries. Similar advantages apply to firms that are listed in one or a few stock markets. Finally, after the financial crisis, the risk-reducing effect of CSR has increased suggesting greater attention to CSR as an instrument to reduce risk by investors. Our analysis of the COVID-19 period shows a strong risk reducing effect for the measures associated with the cost of capital: systematic risk and downside risk.

Our findings have important implications for managers, investors, and policymakers. While managers can reduce risk by undertaking environmental and social investments, investors can use ESG information on firms to reduce information asymmetry, especially in times of financial crises. Furthermore, since the effectiveness of CSR in reducing risk is specific to the institutional setting, policymakers can promote policies to encourage socially responsible actions and benefit from positive environmental and social externalities. Moreover, since the effectiveness of CSR in reducing risk is specific to the institutional setting, policymakers can promote policies to encourage socially responsible actions and benefit from positive both environmental and social externalities. The double benefit is not a detail: our results show that the intensity in risk reducing is the same for both dimensions, environmental and social, even though until now the second one has been neglected.

## Appendix

### Details on ASSET4 sample selection

As LópezPuertas-Lamy et al. (2017) document, ASSET4 produces CSR scores for more than 4,000 firms by evaluating all companies listed in primary market indexes such as CAC 40, DAX, MSCI World, NASDAQ 100, S&P 500, STOXX 600, and others. This process strongly mitigates the risk of selection bias since each firm is evaluated despite its specific CSR and communication strategy. Furthermore, this approach provides a representative global sample of large listed firms, covering a large part of the worldwide market capitalization (Desender et al. 2020). Specialized analysts assess objective key performance indicators (KPIs) and process individual data points from several sources such as stock exchange documents, annual CSR and sustainability reports, nongovernmental organizations, and news websites. Every data point goes through several verification processes to ensure high accuracy. Then a relative weight is assigned to each KPI based on several factors, such as the relevance of the KPI in the industry, whether it is derived from independent information, and its objective measurability. These weighted average scores for each category are adjusted to derive ratings between 0 and 100 for each company and year. Finally, the performance score of the environmental and social pillars is the average of the different category ratings that make up the corresponding pillar, assuming equal weights for each category. The environmental pillar refers to resource reduction, emission reduction, and product innovation benefiting the environment. The social pillar refers to a firm’s employment quality, health and safety, training and development, diversity and opportunity, human rights, community, and customer product responsibility. Given the focus on KPIs, our CSR scores capture the net result of CSR (reflecting concerns and strengths).

### Robustness tests

See Tables 10, 11, 12, 13, and 14.

**Table 10:** Sample breakdown by country, year, and industry

Country	N	%	Country	N	%
Australia	1805	5.45	Luxembourg	46	0.14
Austria	159	0.48	Malaysia	218	0.66
Belgium	282	0.85	Mexico	141	0.43
Bermuda	504	1.52	Netherlands	338	1.02
Brazil	406	1.23	New Zealand	92	0.28
Canada	2040	6.16	Norway	201	0.61
Cayman Islands	335	1.01	Panama	30	0.09
Chile	113	0.34	Papua New Guinea	15	0.05
China	383	1.16	Philippines	106	0.32
Colombia	37	0.11	Poland	122	0.37
Czech Republic	25	0.08	Portugal	106	0.32
Denmark	195	0.59	Qatar	16	0.05
Egypt	60	0.18	Republic of Korea	518	1.56
Finland	286	0.86	Russian Federation	145	0.44
France	1016	3.07	Saudi Arabia	49	0.15
Germany	771	2.33	Singapore	421	1.27
Greece	170	0.51	South Africa	372	1.12
Hong Kong	520	1.57	Spain	435	1.31
India	477	1.44	Sweden	491	1.48
Indonesia	133	0.40	Switzerland	647	1.95
Ireland	187	0.56	Taiwan	743	2.24
Israel	104	0.31	Thailand	98	0.30
Italy	462	1.39	Turkey	158	0.48
Japan	4047	12.22	United Arab Emirates	16	0.05
Jersey	56	0.17	United Kingdom	3076	9.29
Kuwait	32	0.10	United States	9920	29.95
			*Total*	*33,125*	*100*

Year	N	%	Industry	N	%
2002	815	2.46	Basic Materials	3314	10.00
2003	827	2.50	Consumer Goods	3726	11.25
2004	1526	4.61	Consumer Services	4320	13.04
2005	1890	5.71	Financials	6610	19.95
2006	1909	5.76	Health Care	1827	5.52
2007	2046	6.18	Industrials	6148	18.56
2008	2468	7.45	Oil and Gas	2377	7.18
2009	2841	8.58	Technology	2360	7.12
2010	3358	10.14	Telecommunications	843	2.54
2011	3381	10.21	Utilities	1600	4.83
2012	3347	10.10	*Total*	*33,125*	*100*
2013	3292	9.94			
2014	3233	9.76			
2015	2192	6.62			
*Total*	*33,125*	*100*

**Table 11:** Country-level contingency analysis

	$\mathtt {CommonLaw}$	$\mathtt {CivilLaw}$	$\Delta$	$\mathtt {HighSecReg}$	$\mathtt {LowSecReg}$	$\Delta$	$\mathtt {HighDisReq}$	$\mathtt {LowDisReq}$	$\Delta$
*Panel A-Legal environment*									
$\sigma$	0.0018	− 0.0237***	0.0255**	0.001	− 0.0134*	0.0144	-0.0004	− 0.0115*	0.0111
	[0.0064]	[0.0082]	[0.0104]	[0.0074]	[0.0072]	[0.0103]	[0.0078]	[0.0069]	[0.0104]
$\beta$	− 0.0519**	− 0.1198***	0.0679	− 0.033	− 0.1133***	0.0803*	− 0.0210	− 0.1138***	0.0928**
	[0.0249]	[0.0354]	[0.0433]	[0.0278]	[0.0315]	[0.042]	[0.0289]	[0.0298]	[0.0415]
$\beta ^{-}$	− 0.0207	− 0.1643***	0.1436***	− 0.0098	− 0.1303***	0.1205**	0.0026	− 0.1380***	0.1406***
	[0.0303]	[0.0404]	[0.0505]	[0.0331]	[0.0364]	[0.0492]	[0.0342]	[0.0346]	[0.0486]
$\sigma _{\epsilon }$	− 0.0002	− 0.0202***	0.0200**	− 0.0027	− 0.0117*	0.009	− 0.0039	− 0.0105*	0.0066
	[0.0059]	[0.0071]	[0.0092]	[0.0067]	[0.0063]	[0.0092]	[0.0070]	[0.0060]	[0.0092]

	$\mathtt {LowAemScore}$	$\mathtt {HighAemScore}$	$\Delta$	$\mathtt {HighIrFreq}$	$\mathtt {LowIrFreq}$	$\Delta$			
*Panel B-Financial information environment*									
$\sigma$	0.0026	− 0.0221***	0.0247**	0.0046	− 0.0145*	0.0191*			
	[0.0069]	[0.0082]	[0.0107]	[0.0079]	[0.0075]	[0.0109]			
$\beta$	− 0.0630**	− 0.1160***	0.053	− 0.0289	− 0.1454***	0.1165***			
	[0.0271]	[0.0357]	[0.0448]	[0.0296]	[0.0333]	[0.0446]			
$\beta ^{-}$	− 0.0427	− 0.1531***	0.1104**	− 0.0074	− 0.1769***	0.1695***			
	[0.0331]	[0.0399]	[0.0518]	[0.0350]	[0.0381]	[0.0517]			
$\sigma _{\epsilon }$	0.0029	− 0.0220***	0.0249***	0.0017	− 0.0094	0.0111			
	[0.0063]	[0.0070]	[0.0094]	[0.0072]	[0.0064]	[0.0096]			

**p*<0.10, ***p*<0.05, ****p*<0.01

The table shows results concerning the moderating role of the legal (Panel A) and financial information (Panel B) environments on the relationship between CSR and equity risk. Firms are divided into subgroups according to their country characteristics. Panel A focuses on the common/civil-law divide, on the level of security regulation and on the level of disclosure requirements. Panel B focuses on the level of earnings management and the interim reporting frequency. Only the coefficient $\theta$ of each model (one for each equity risk measure and country-level division) is reported in the table together with the subgroup difference test ($\Delta$). Estimates are based on the fixed-effects estimator. Inference is based on cluster robust standard errors, reported in square brackets

**Table 12:** Industry and firm level contingency analysis

	$\mathtt {HighImpact}$	$\mathtt {LowImpact}$	$\Delta$	$\mathtt {HighProfile}$	$\mathtt {LowProfile}$	$\Delta$	$\mathtt {HighCl}$	$\mathtt {LowCl}$	$\Delta$
$\sigma$	− 0.0223**	− 0.0059	− 0.0164	0.0005	− 0.0133**	0.0138	− 0.0109	− 0.0067	− 0.0042
	[0.0113]	[0.0057]	[0.0127]	[0.0083]	[0.0064]	[0.0105]	[0.0068]	[0.0085]	[0.0109]
$\beta$	− 0.0387	− 0.0798***	0.0411	− 0.0211	− 0.1001***	0.0790*	− 0.1268***	0.0094	− 0.1362***
	[0.0476]	[0.0237]	[0.0532]	[0.0353]	[0.0262]	[0.044]	[0.0291]	[0.0332]	[0.0441]
$\beta ^{-}$	− 0.0616	− 0.0730***	0.0114	− 0.0366	− 0.0891***	0.0525	− 0.1206***	0.0035	− 0.1241**
	[0.0561]	[0.0276]	[0.0625]	[0.0423]	[0.0308]	[0.0523]	[0.0335]	[0.0401]	[0.0523]
$\sigma _{\epsilon }$	− 0.0212**	− 0.0061	− 0.0151	− 0.0016	− 0.0122**	0.0106	− 0.0063	− 0.0156**	− 0.0474***
	[0.0102]	[0.0051]	[0.0114]	[0.0074]	[0.0057]	[0.0093]	[0.0060]	[0.0077]	[0.0098]

**p*<0.10, ***p*<0.05, ****p*<0.01

The table shows results concerning the moderating role of industry and firm characteristics on the relationship between CSR and equity risk. Firms are divided according to whether they belong to industries with a high or low impact on stakeholders, industries with a high or low profile, and firms with a number of cross-listings above or below the median. Only the coefficient $\theta$ of each model (one for each equity risk measure and industry-level o firm-level division) is reported in the table together with the subgroup difference test ($\Delta$). Estimates are based on the fixed-effects estimator. Inference is based on cluster robust standard errors, reported in square brackets

**Table 13:** Contingency analysis by time

	2002–2006	2007–2009	2010–2015
$\sigma$	0.0008	0.0051	− 0.0033
	[0.0096]	[0.0111]	[0.0077]
$\beta$	− 0.0539	− 0.0456	− 0.0520*
	[0.0544]	[0.0344]	[0.0271]
$\beta ^{-}$	− 0.0362	− 0.1589***	− 0.0278
	[0.0797]	[0.0474]	[0.0380]
$\sigma _{\epsilon }$	0.0027	− 0.0050	− 0.0078
	[0.0086]	[0.0097]	[0.0070]

**p*<0.10, ***p*<0.05, ****p*<0.01

The table shows results concerning the moderating role of the financial-crisis phase on the relationship between CSR and equity risk. Observations are divided according to whether they pertain to the pre-, during- or post-crisis periods (i.e., 2002–2006, 2007–2009, 2010–2015). Only the coefficient $\theta$ of each model (one for each equity risk measure and period) is reported in the table. Estimates are based on the fixed-effects estimator. Inference is based on cluster robust standard errors, reported in square brackets

**Table 14:** CSR effects on risk measures from 2016–2020

	$\sigma$	$\beta$	$\beta ^{-}$	$\sigma _{\epsilon }$
*Panel A-Country-level contingency analysis: Legal environment*				
$\mathtt {ES}_{t-1}\times \mathtt {CommonLaw}$	0.0025***	− 0.0034**	0.0004	0.0013***
$\mathtt {ES}_{t-1}\times \mathtt {HighSecReg}$	0.0019***	− 0.0027**	− 0.0037*	0.0011***
$\mathtt {ES}_{t-1}\times \mathtt {HighDisReq}$	0.0016***	− 0.0027**	− 0.0039*	0.0008***
*Panel B-Country-level contingency analysis: Financial information environment*				
$\mathtt {ES}_{t-1}\times \mathtt {HighAemScore}$	− 0.0017***	0.0022	0.0010	− 0.0007***
$\mathtt {ES}_{t-1}\times \mathtt {HighIrFreq}$	0.0015***	− 0.0028**	− 0.0029	0.0007**
*Panel C-Industry-level contingency analysis*				
$\mathtt {ES}_{t-1}\times \mathtt {HighImpact}$	− 0.0001	0.0013	0.0028	− 0.0004
$\mathtt {ES}_{t-1}\times \mathtt {High Profile}$	− 0.0001	0.0007	0.0039*	0.0001
$\mathtt {ES}_{t-1}\times \mathtt {Cl}$	0.0005	0.0022	0.0039**	0.0000

**p*<0.10, ***p*<0.05, ****p*<0.01

This table shows interaction models for the equity risk measures total risk ($\sigma$), beta ($\beta$), downside beta ($\beta ^{-}$) and idiosyncratic risk ($\sigma _{\epsilon }$). We report the coefficients on the lagged CSR measure (based on the new Refinitiv methodology) interacted with the country-level contingency variables (Panels A and B) and the industry-level contingency variables (Panel C). Estimates are based on the two-step Arellano and Bover (1995)/Blundell and Bond (1998) GMM system estimator. Inference is based on Windmeijer (2005) robust standard errors

## Footnotes

1 Analyzing ownership structure, Chang et al. (2021) focus on the role of local institutional shareholders for the level of CSR activities, while Roberts (1992) discusses the role of stakeholder beyond owners. Jackson and Apostolakou (2010) analyze sectoral differences in the impact on stakeholders. Boubakri et al. (2016) investigate the dynamics of cross-listing and CSR.

2 While most previous studies point towards risk mitigation through CSR, other papers strongly disagree. Under an agency view of CSR, managers will have a tendency to wastefully pursue their own interests at the expense of shareholders (Friedman 1970; Tirole 2001; Jensen 2002). Instead of maximizing shareholder value, corporate managers may use CSR to improve their own reputation, to foster their political or career agendas, to create an impression of ethical behavior to mask corporate misconduct (Hemingway and Maclagan 2004) or for earnings management (Prior et al. 2008). Clearly, wasteful CSR activities would not translate into lower equity risk.

3 Total risk can be decomposed into its systematic and idiosyncratic components using $\sigma ^{2}_{i}=\beta ^{2}_{i}\sigma ^{2}_{m}+\sigma ^{2}_{\epsilon ,i}$.

4 The measure is based on an index of disclosure requirements in securities offerings.

5 Datastream does not provide time-series information on cross-listings. The value available to us is for the calendar year 2017.

6 Calculated as ($$-0.0139 \frac{0.29}{0.15\cdot (1-0.4075)}=-0.05$$).

7 Note that in some cases the coefficients of subsamples are not significant, while the corresponding coefficient in Table 2 is statistically significant. The reason is that while GMM allows us to deal with autocorrelation and endogeneity, this flexibility comes at a price: due to sample variability, some variables of interest lose significance in the contingency analysis. However, variables that are significant are consistent with the main analysis reported in Table 2. Further, we provide a robustness check in the Appendix, using the fixed-effects estimator. The results shown in Table 11 are qualitatively similar to those in Table 3, but with stronger statistical significance.

8 However, we provide results using the fixed-effects estimator in Table 12 in the Appendix.

9 In Table 13 in the Appendix, we provide results for the fixed-effects estimator . Results are qualitatively similar but with weaker statistical significance.

10 While the Hansen (1982) test is an alternative to the Sargan test when heteroscedasticity occurs, many instruments, as in our application, strongly weaken and make it unreliable. To save space e improve clarity, we tabulate the results on the Arellano-Bond and Sargan specification tests only for Table 2, but they are available upon request for all the estimated models in the paper.

11 In Table 14 of the Appendix, we provide results on the contingency analysis with recent data. Given the new rating methodology that conditions scores on intra-industry and intra-country comparison groups, it is not surprising that results are not as clear cut as in our main analysis.
